# The effects of electrical stunning on the nervous activity and physiological stress response of a commercially important decapod crustacean, the brown crab *Cancer pagurus* L.

**DOI:** 10.1371/journal.pone.0270960

**Published:** 2022-07-26

**Authors:** Douglas M. Neil, Amaya Albalat, John Thompson

**Affiliations:** 1 Institute of Biodiversity, Animal Health and Comparative Medicine, School of Medical Veterinary and Life Sciences, University of Glasgow, Glasgow, Scotland, United Kingdom; 2 Institute of Aquaculture, University of Stirling, Stirling, Scotland, United Kingdom; COISPA Tecnologia & Ricerca - Stazione Sperimentale per lo Studio delle Risorse del Mare, ITALY

## Abstract

Increasing attention is being paid to the welfare of decapod crustaceans. Legislation exists for their humane slaughter in several countries and this is being debated in others. Electrical stunning may have potential for humane slaughter of crustaceans in some circumstances, although scientific data on the effectiveness of electrical stunning when applied to various species are limited. Assessment criteria for effective stunning have so far been based mainly on behavioural assessments, but these do not always reflect neural insensibility. In this study direct recordings of neural activity, both centrally and peripherally, have been used to provide more direct measures of the state of sensibility. We have also examined whether electrical stunning acts as a physiological stressor, using measures of haemolymph L-lactate. Experiments were performed on a commercially important decapod species, the brown crab *Cancer pagurus* L. Spontaneous activity within the CNS was arrested by electrical stunning, which is an indication of loss of sensibility. There were also specific effects on the peripheral nervous system, with loss of responsiveness to sensory stimulation, rendering the animals unresponsive to external stimuli, and a failure of motor activation. All these effects were apparent immediately after a 10s stun, and persisted for as long as tested (4h) indicating that the animals were also killed by the procedure. No autotomy of limbs occurred. Haemolymph L-lactate was found to be no greater following electrical stunning than after handling and sampling alone, and both were significantly lower than values reached in a range of environmental and commercial situations. For all these reasons we find that electrical stunning may meet criteria for humane slaughter of *C*. *pagurus*.

## Introduction

Today in several western countries increased attention is being paid to the welfare of decapod crustaceans at all stages of their capture, holding and use for both commercial and scientific purposes. The term welfare is intrinsically linked with the absence of suffering, pain and distress, and according to most national and international policy frameworks only applies to animals that are sentient. There is considerable evidence for decapods being sentient [1, 2], although this has been questioned by some authors [3]. However, applying a ‘precautionary principle’ [4], or simply through concern for all animals ‘in our care’ [5, 6] a requirement to use more humane methods of slaughter is now demanded by advocacy bodies [7, 8]. This is increasingly being mandated by legislative bodies and governments in several parts of the world [9–11], for example, decapods have been recognized as being sentient by the parliament of the United Kingdom https://www.gov.uk/government/news/animals-to-be-formally-recognised-as-sentient-beings-in-domestic-law.

Various methods have been used to kill decapod crustaceans prior to marketing, transport or consumption. These include freezing, superchilling (N_2_ gas), salt baths (MgCl_2_), asphyxiation with CO_2_, chilling in air or ice slurry, or boiling [12, 13]. However, despite evidence that some traditional dispatch methods can result in rapid (<10 s) and irreversible death in some species of small crustaceans [14], when applied to large decapod crustaceans the majority of these methods are considered inhumane by the European Union’s Scientific Panel on Animal Health and Welfare [15] since most take some time to have an effect and hence have the potential to confer suffering or stress.

Methods of dispatch that minimise the potential to inflict suffering and stress, and thus improve welfare, continue to be sought by the crustacean fishery and aquaculture industries [16–18]. These include an initial effective anaesthetisation before killing [19–21] or a rapid stunning, either mechanical or electrical, that induces immediate insensibility by inactivating the nervous system [16, 22, 23]. Electrical stunning is used widely in the slaughter of domesticated farm animals [24], and can also be effective on aquatic animals such as fish [25, 26]. It therefore seems to offer potential for humane killing of decapod crustaceans, and indeed a number of stunning devices have been developed for this purpose: LAVES (adapted from a fish stunner) [27], STANSTAS [28] and CRUSTASTUN™ [16, 27, 29]. In some studies, equivalent laboratory-constructed equipment has been used [30, 31]. For each of these devices the optimal electrical parameters for stunning large decapod crustaceans (crabs, lobsters, crayfish) have been determined. More generally, these parameters include waveform, minimum current and voltage, maximum frequency and minimum time exposure [32]. The presence of a thick, hard exoskeleton in these large crustaceans means that the required voltage needs to be higher, compared to prawns and shrimps.

The methods for monitoring insensibility in crustaceans have most often been based on behavioural observations such as body posture, appendage movement or reflex reactions of the eyes and antennae. In some cases, for the purpose of comparing different slaughter methods, these observations have also been incorporated into numerical behavioural scores or ‘vitality’ indices [28, 30, 33, 34]. However, the difficulty with using such behavioural criteria to assess stunning effectiveness is that they are subject to misinterpretation, since electrical stunning often induces immobility (an inability of the muscle to contract) or epileptic seizure [27, 31], which makes it impossible to judge if complete neural insensibility has actually occurred. Only a few studies have monitored nerve activity directly after electrical stunning of decapod crustaceans. Fregin and Bickmeyer (2016) used implanted electrodes alongside the abdominal nerve cord of lobsters to measure changes in mass discharge within the central nervous system while testing various possible anaethetising methods, including electrical stunning [27], and Weineck et al (2018) monitored the heart rate in crabs, crayfish and shrimp during electroshocking or cold shock [31]. In neither case, however, was it determined whether there was also suppression of either the sensory or motor signals within the peripheral nervous system, which is highly relevant information for determining whether a state of insensibility is effectively induced by electrical stunning. A related question is whether electric stunning by itself can act to accomplish ‘simple stunning’ (i.e. irreversible death) of decapod crustaceans [6], or whether it should be regarded as an anaesthetic that by itself is not lethal, but renders the animal insensible for a sufficient time to allow other procedures to effect humane slaughter.

Additionally, it is important to determine if the electrical parameters used for effective stunning act as physiological stressors, inducing stress responses that may affect body condition and thus leading to a reduced welfare. Stress is a physiological response that is distinct from pain/nociception [35] and involves primary and secondary responses that attempt to rebalance the homeostasis of the body [11, 36]. Thus, for example, the crustacean hyperglycaemic hormone (CHH) mediates primary stress responses [37–39], while L-lactate is involved in secondary responses, being the major end product of anaerobic metabolism. Higher haemolymph concentrations of L-lactate indicate an attempt by the animal to mitigate the effect of a variety of stressors: locomotion [40]; emersion [41]; de-clawing [42]; trawling [43, 44]; post-capture handling and transport [38, 45]. Also of relevance is a report that mild electric shocks can result in increased circulating L-lactate [46] which may indicate stress and/or a physiological response to muscular contraction. Therefore, monitoring this metabolite during and after electric stunning may provide a way to assess the relative stressfulness of this procedure.

The present study provides some of the relevant neurophysiological data necessary to establish the effectiveness of the electrical stunning delivered using a commercial instrument in decapod crustaceans. This has been achieved by recording neuronal activity at various points along the sensory-CNS-motor chain, allowing changes in particular parts of the central and peripheral nervous systems to be distinguished. For some purposes recordings have been made from isolated limbs, exploiting the natural process of autotomy. This also conveniently allows the effectiveness of motor nerve stimulation to produce muscle contraction to be evaluated. Because electrical stunning may not always kill crustaceans outright [27, 30], the ability of the procedure to achieve ‘simple stunning’ has been assessed by monitoring for several hours after the stun. The possibility that electrical stunning imparts physiological stress has also been evaluated by measuring levels of L-lactate circulating in the haemolymph. The species used in this study was the European edible crab or brown crab (*Cancer pagurus* L.), a commercially important brachyuran decapod crustacean that is exploited throughout Western Europe [47]. It is the most valuable crab fishery in UK waters [48], being worth £11.2 million in 2020 in Scotland alone [49]. This species is commonly supplied live to processors and to the restaurant trade in the UK and other European countries, and for this reason it has already been used in studies of electrical stunning [28, 30, 33].

## Materials and methods

### Anatomy

**Fig 1:**
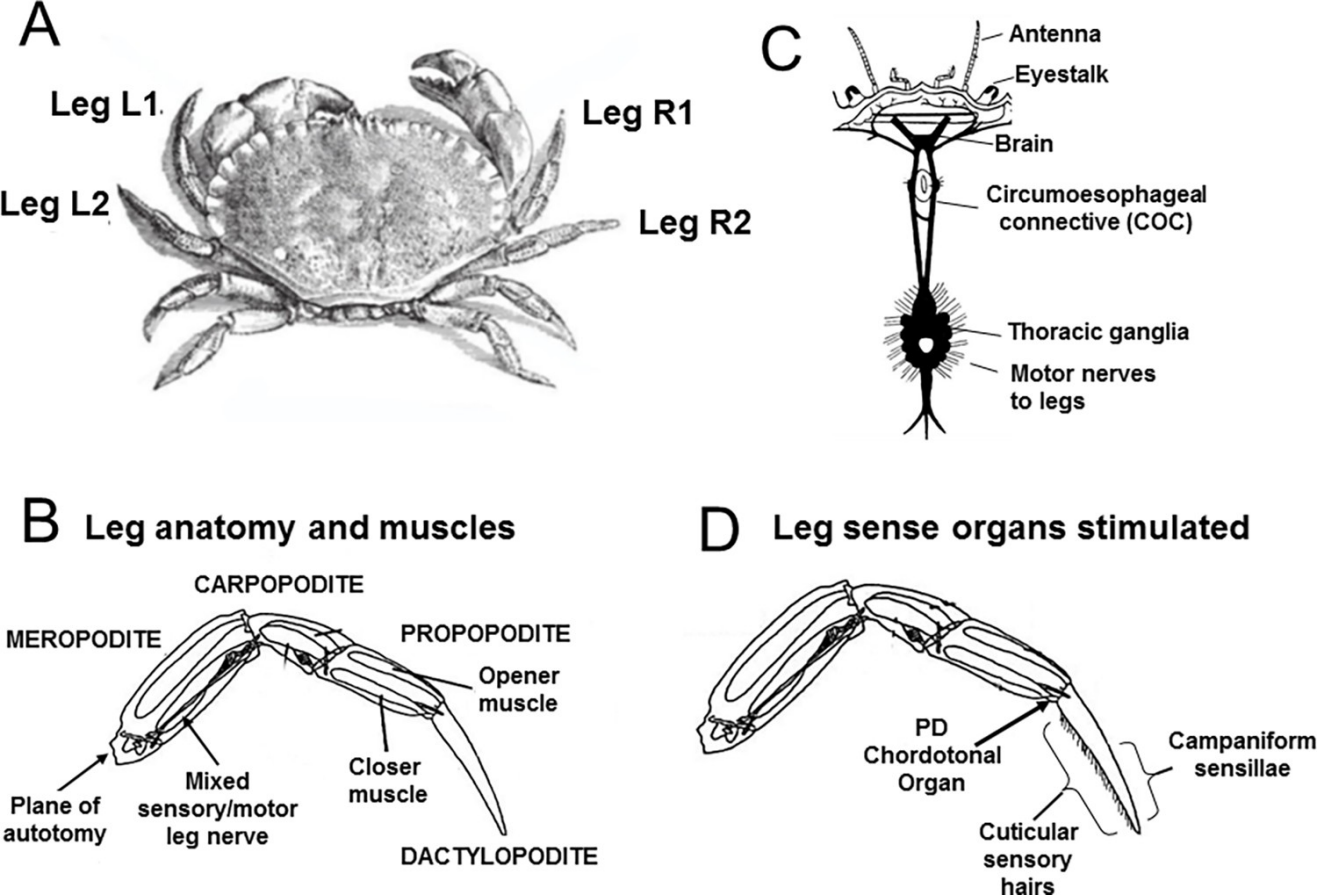
Schematic diagram of the anatomy of the crab *Cancer pagurus* L. A, External anatomy and numbering of legs. B, Arrangement of the nervous system. C, Leg anatomy and muscles. D, Leg sense organs stimulated in the current study.

Decapod crustaceans, the taxonomic group to which crabs belong (external anatomy shown in Fig 1A), have nervous systems of the characteristic arthropod plan with paired nerve cords, a dorsal brain (supraoeophageal ganglia) and separate circumoesophageal connectives [50] which carry neuronal traffic both to and from the brain. In crabs a distinct abdomen has been reduced and the thoracic ganglia are condensed into a single thoracic mass, from which all the peripheral nerve roots emerge (Fig 1B). The axons of both the sensory and motor neurones pass in mixed leg nerves that travel through the centre of the leg segments.

Each of the four pairs of walking legs (pereiopods) of crabs comprises a series of articulated segments, which are moved by paired muscles (Fig 1C). A number of different mechanoreceptors are associated with the leg exoskeleton (Fig 1D), including innervated cuticular sensory hairs that signal contact and water movement [51], and a type of campaniform sensilla (‘funnel canal organs’) which are pressure-sensitive [52]. In addition, a series of elastic strands span the various joints, into which are embedded sensory cells which detect joint flexion and extension [53]. These so-called chordotonal organs thus act as proprioceptors monitoring the leg movements made by the crab [54]. The chordotonal organ spanning the terminal leg segment, between the propopodite and the dactylopodite (the PD chordotonal organ) [55] was selectively activated in this study.

### Ethical statement

Ethical approval for procedures on decapod crustaceans is currently not required in the UK. Nevertheless, all the live crabs used were treated with proper care in order to minimise their discomfort and distress. There was no practical alternative to the use of live animals. The total number of crabs used (n = 30) was the minimum required to obtain a reasonable threshold of certainty for statistical validation, considering that the gain in knowledge and long term benefit to the subject will be significant. Prior to experimentation they were housed communally to ensure behavioural enrichment. Experimental design considered the 3R’s (Replacement, Reduction and Refinement). Minimisation of suffering and distress was inherent to the experimental design in that the objective was to render the crabs unconscious by electrical stunning prior to, if necessary, inducing death. No anaesthesia was used. The exposure of subjects to experimental treatment did not exceed 10 s. To ensure welfare, behaviour of experimental subjects was monitored for indications of consciousness recovery every 15 min following stunning. No crabs had to be euthanased; all were killed by the electrical stunning treatment while unconscious. Therefore, the pre-defined humane endpoint (consistent behavioural indications of consciousness recovery post stunning) was never invoked.

### Animal supply and holding

Male brown crabs, *Cancer pagurus* L. of carapace width 120–140 mm, were used. The crabs were captured by commercial fishermen using baited traps (creels) laid offshore from St Abbs on the east coast of Scotland in the spring of 2012. All animals were in the intermoult stage with a hard exoskeleton. Initially they were held communally in seawater tanks at the St Abbs Marine Station, and were then transferred in chilled containers to the University of Glasgow. Here they were retained individually in tanks within a closed seawater circulating system at 10°C for at least two weeks before experimentation. From this stock 18 crabs were subjected to electrical stunning for either behavioural evaluation, electrophysiological recording or measurements of stress, 6 crabs were used in sham trials and another 6 used as controls.

### Electrical stunning

The Crustastun^™^ machine (Studham Technologies, UK) is a dry/in air device designed to administer a lethal electric shock to individual decapod crustaceans such as crabs and lobsters before dismemberment and cooking, in order to avoid boiling a live animal. Within the device an electrical charge of 110 volts, 2–5 amps is applied to a single animal across two electrodes for 5–10 s. These parameters were determined by Robb in 1999 and the effectiveness of the Crustastun^™^ on crabs was evaluated by Sparrey in 2005, who found that the target current of 1.3 amp was achieved within 0.5 s, was maintained throughout the stun cycle, but fell to <0.5 amp within 1 s of its termination [16].

The electrical stunning procedure was applied without prior anaesthesia of the crabs, using the Crustastun™ machine according to the manufacturer’s operating instructions. The chamber was filled with a salt solution (~3g L^-1^). Individual crabs were removed from their holding tanks and placed into the Crustastun™ machine, the lid was closed and the animal was stunned by a 110 volt, 2–5 amp electrical charge for 10 s. The animal was then returned to its seawater container (water temperature 10–12°C).

### Behavioural measures

A qualitative assessment of the behavior and movements of a subset of 6 crabs was carried out before and after electrical stunning, to provide a preliminary evaluation of their vigour, activity and responsiveness. Movements of the whole animal and of the limbs, mouthparts and antennae were noted, and reflex eyestalk withdrawal was induced by touching the anterior carapace.

### Exposing the nervous system

Crabs not subjected to electrical stunning were held on ice for 30 min prior to experimentation to induce torpor, while those subjected to electrical stunning were used directly after the stun. To expose the central nervous system of the crab for electrophysiological procedures the carapace was removed and the preparation was submerged in a balanced salt solution corresponding in composition and osmolarity to crab haemolymph, at a temperature of 10°C. The internal organs were then removed or displaced in order to expose the circumoesophageal connectives around the base of the stomach.

Isolated legs were prepared for nerve recording and stimulating by inducing intact crabs to shed a leg spontaneously (autotomy) by applying pressure to the basipodite segment [56, 57]. In some cases, to determine the longevity of nerve activity, two legs were autotomised from one crab at the same time, and while one was dissected and tested immediately the other was held in saline for up to 4 h before being prepared for recording. The same procedure was followed for the electrically stunned crabs, except that the legs were detached by amputation (since these crabs did not express the autotomy reflex).

In all cases the M-C joint between the meropodite and carpopidite was then disarticulated, the muscle tendons spanning this joint were cut and the leg was separated at this point with the leg nerve still attached to the distal portion. The leg nerve was teased into a number of separate bundles, to expose the axons and to facilitate selective recording or stimulation. This isolated leg preparation was then submerged in physiological saline at 10°C until required.

### Electrophysiological recordings

Electrophysiological recordings were made from the exposed nerves using various extracellular techniques. For recording both ascending and descending neuronal traffic from the circumoesophageal connectives a suction electrode method was used in an ‘*en passant’* configuration [58]. A fine-tipped polythene electrode containing physiological saline was applied to the surface of the intact nerve, and a gentle suction was applied through attached tubing and a syringe. In some cases, neuronal traffic in the ascending or descending directions was recorded separately by cutting the circumoesophageal connective and applying the suction electrode to either the anterior or posterior cut end of the nerve.

**Fig 2:**
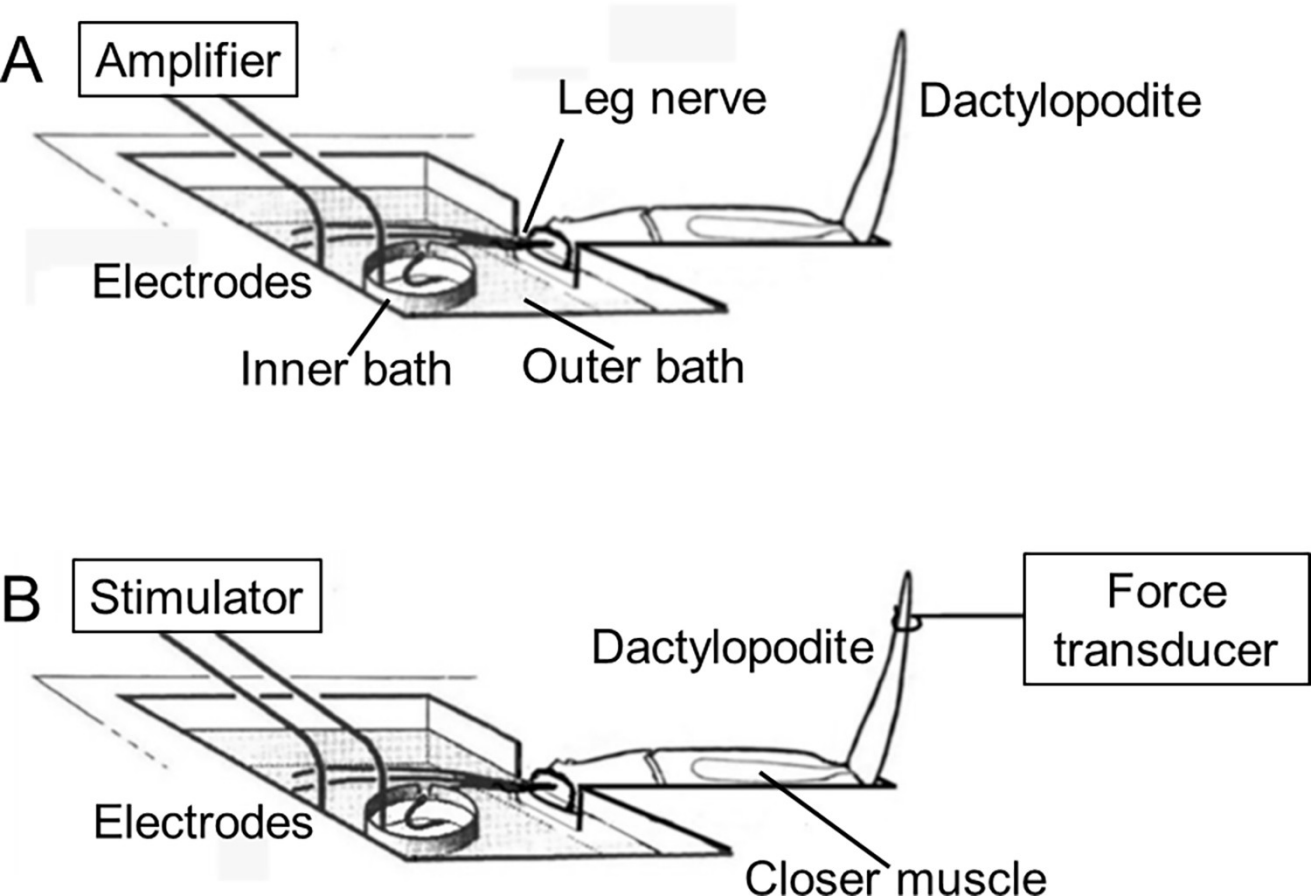
Experimental arrangements. (A) Recording from the nerve of an isolated *C*. *pagurus* leg. (B) Stimulating the crab leg nerve while recording the forces produced by the dactylopodite closer muscle.

For recording sensory activity from the leg nerve, an isolated leg preparation was clamped to a plastic plate and a nerve bundle was passed from an adjacent outer bath through a wall of petroleum jelly into a second small inner bath, both of which contained physiological saline (Fig 2A). A bipolar electrode of two silver wires was used to make contact with the solutions in the inner and outer chambers respectively.

For both the central and peripheral recordings the signals from the extracellular electrodes were passed to a differential pre-amplifier (A101, Isleworth Ltd.) for amplification and filtering. The amplifier output was then passed to an Analog/Digital converter (PowerLab, AD Instruments Ltd) and was both displayed and recorded on a standard PC computer using the associated software (Chart v7, AD Instruments Ltd.)

### Recording muscle force

Direct recordings of motor neurone activity were not attempted, due to the relative inaccessibility of the motor roots emerging from the thoracic ganglion in *C*. *pagurus*.

Instead, the state of activation of the motor pathways was ascertained using the isolated leg preparation by stimulating the nerve bundles (containing motor nerves) at various frequencies while monitoring the force produced by the dactylopodite closer muscle. For this purpose one or more nerve bundles were passed from the outer bath to the inner bath (as above), and the bipolar electrode making contact with solution in the two chambers was now connected to an isolated stimulator within the PowerLab (Fig 2B). Patterns of stimulating pulses at various amplitudes and frequencies were applied using a software ‘stimulator control panel’ within the Chart v7 software. Typically, stimulus trains of 3 s duration and 4 V amplitude were applied at a range of frequencies from 10–100 Hz.

Although stimulation of the leg nerve bundles potentially activated motor neurones supplying several of the muscles located more proximally in the leg, the forces produced by the closer muscle of the terminal propopodite/dactylopopodite joint (P-D) were nevertheless measured selectively. This was achieved by cutting the tendon of the antagonist muscle about that joint (the P-D opener muscle), and then attaching a thread from near the tip of the dactylopodite to the arm of a sensitive force transducer (FT-03, Grass Instruments Ltd.), mounted on a micromanipulator (Fig 2B). This selectively monitored the forces produced by the dactylopodite closer muscle. The output of the transducer was passed to a custom-built amplifier (x1000), and then fed to an input of the Powerlab A/D converter. The forces and the stimulus parameters were then both displayed and recorded on a standard PC computer using the Chart v7 software.

### Stress measurement

From the stock of crabs being held, two groups of 6 were chosen randomly from the holding tanks and were subjected to either the electrical stunning procedure or a sham treatment in which the animals were handled in exactly the way, but not stunned. This sham treatment was used to provide a control for the effects of the handling which inevitably occur prior to and after the stunning procedure.

For a crab to be electrically stunned an initial haemolymph sample was taken for L-lactate determination (pre-stun value), then the crab was placed into the Crustastun™ machine, the lid was closed and the animal was stunned by a 110 volt, 2–5 amp electrical charge for 10 s. The crab was then returned to a seawater tank (water temperature 10–12°C). This entailed the crab being emersed into the air for no more than 2 minutes. A second haemolymph sample (from the contralateral side) was taken at a time point of 10 minutes after the electrical stunning procedure, for another L-lactate determination (post-stun value). No further samples were taken at later time points as it was found that the crabs were effectively killed by the electrical stunning procedure, and were then in a post-mortem state.

For a crab receiving the sham treatment an initial haemolymph sample was taken for L-lactate determination (pre-sham value), then the crab was then placed into the Crustastun™ machine and the lid was closed for 10 s, but without activation of the electrical charge. The crab was then returned to its seawater tank (water temperature 10–12°C). As with stunning procedure, the crab was emersed into the air for no more than 2 minutes. A second haemolymph sample (from the contralateral side) was taken at a time point of 10 minutes after the sham treatment, for L-lactate determination (post-sham value). Also, in order to gauge recovery, a further haemolymph sample was taken 24 h later.

### Haemolymph sampling and measuring L-lactate

Haemolymph samples were taken from the sinus at the base of a 5th pereiopod using a 25-gauge needle and a disposable syringe. The L-lactate concentration was measured in the haemolymph samples with a portable lactate analyser (Accutrend ®, Roche Diagnostics, Basel, Switzerland) using freshly extracted samples. The accuracy of the portable lactate analyser for the determination of L-lactate in decapod crustacean haemolymph samples had previously been determined by analysing a set of haemolymph samples from the Norway lobster using an enzymatic method [17] and comparing these values with those obtained using the lactate analyser. It was found that there was a highly significant correlation (r^2^ = 0.960) between the values for haemolymph L-lactate obtained using the two methods.

### Statistical analysis

Statistical analyses were carried out for each measure by a General Linear Model (GLM), treating stunned and sham-treated crabs as separate experiments. The response variable was the haemolymph L-lactate concentration measure and the explanatory variable was the treatment (as a categorical factor). The residuals were assessed visually for normality.

Data are reported as mean values ± standard error of mean (SEM). Pairwise comparisons were analysed by independent samples t-tests. Data were tested for normality using the Levene’s test. P-values lower than 0.05 were considered statistically significant (SPSS, Statistics version 23).

## Results

The trials involved a total of 18 individual crabs that were electrically stunned and 12 intact crabs as control and sham groups. In the case of isolated legs, up to three legs per individual were tested. The neuronal data are presented as traces of the original electrophysiological recordings and where appropriate also as plots of the muscle forces produced in response to the varying strength of stimulus.

### Behavioural evaluation

In order to relate the electrophysiological data obtained here to other published studies on electrical stunning which used behavioural criteria for evaluation [28, 30, 33], we initially made a qualitative assessment of the behavior and survival of a group of 6 crabs at different times following electrical stunning. Immediately after the short-duration (10 s) electrical stun all of these crabs showed no further visible activity (limb movement, antennule flicking, mouthpart movement, ventilation current and eye retraction reflexes), and never showed any signs of recovery up to the longest time observed (4 h). We interpreted this as indicating that they were effectively killed. Moreover, one feature that was never observed as a result of electrical stunning was an induced autotomy of either the claws or the walking legs.

### Activity in the central nervous system of intact crabs

**Fig 3:**
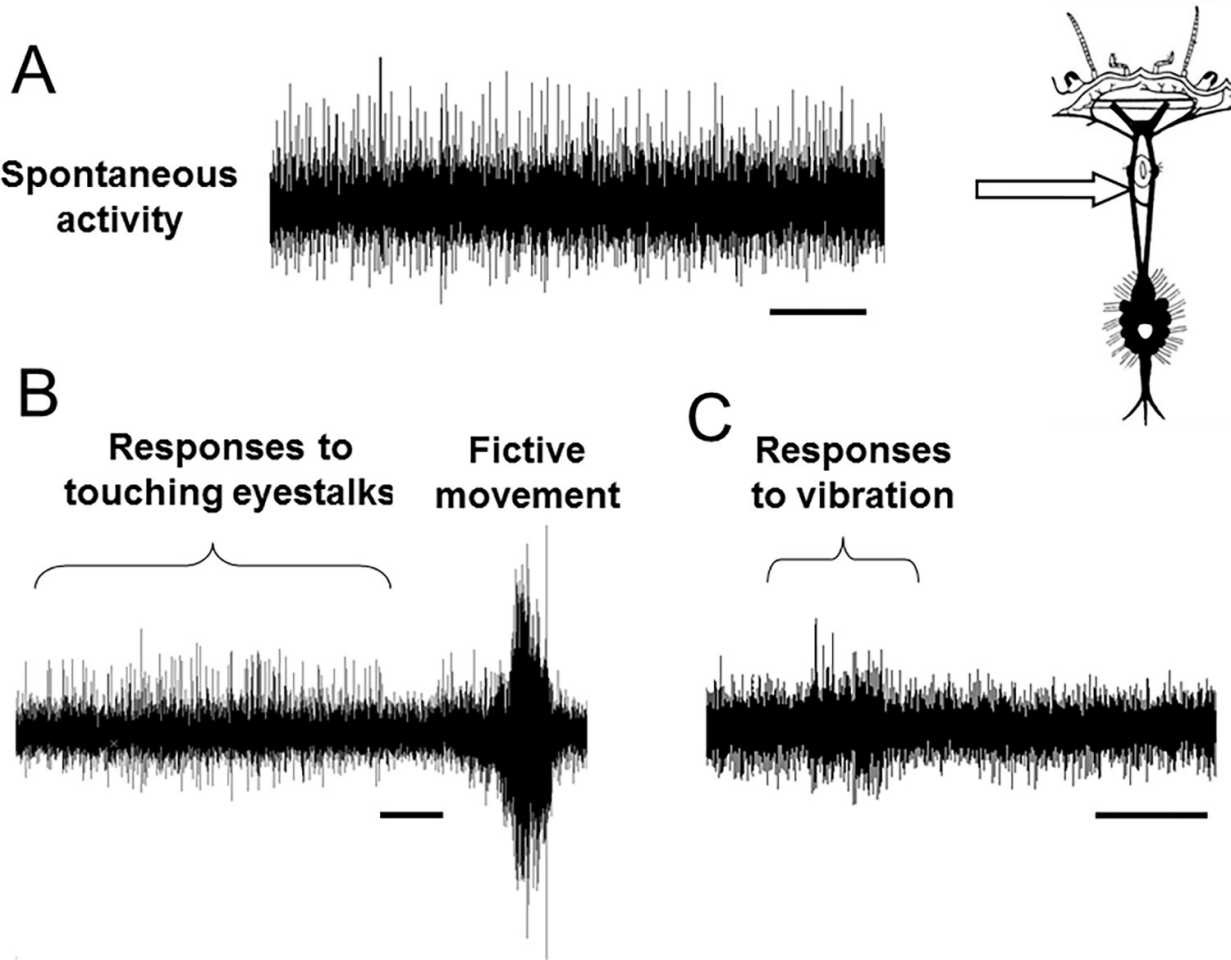
Recordings from the central nervous system in intact crab. Nerve activity recorded extracellularly in the left circumoesophageql connective of an intact *C*. *pagurus* crab. A, Spontaneous activity; B, Responses to touching eyestalks and a burst associated with fictive locomotion; C, Responses to vibration. Scale bars 2.5 s.

Recordings made from one or both circumoesophageal connectives in intact crabs indicated that there was a high level of spontaneous neuronal activity passing along the axons of these nerve bundles, even in the absence of any imposed stimulation (Fig 3A). The variety of sizes of the extracellularly-recorded spikes indicate that the signals arose from a large number of different individual nerve axons of varying diameters.

**Fig 4:**
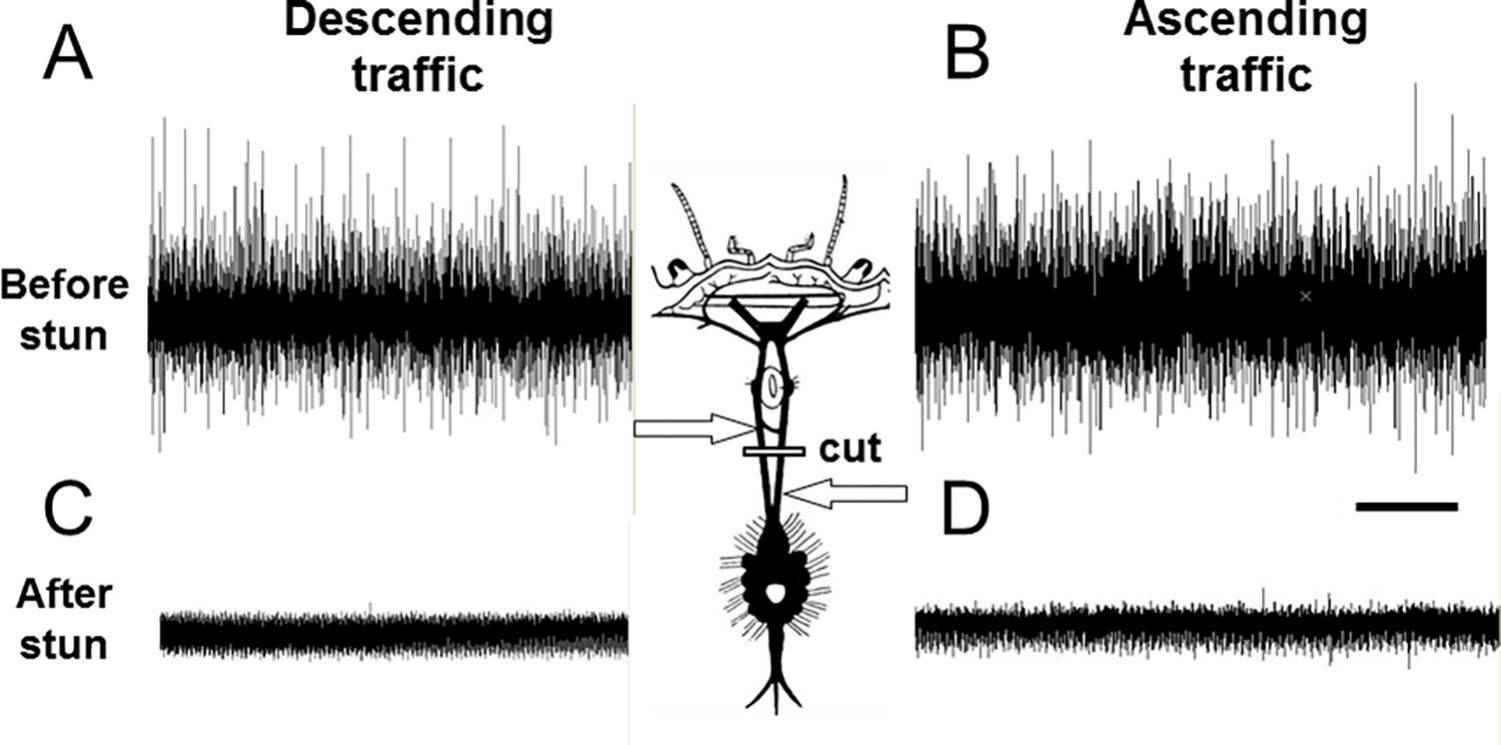
Recording CNS activity before and after electrical stunning. Spontaneous nerve activity recorded extracellularly in the left and right circumoesophageal connectives of *C*. *pagurus* after cutting at the level indicated. A,B: intact animals; C,D: 10 min after electrical stunning. Scale bar 1s.

**Fig 5:**
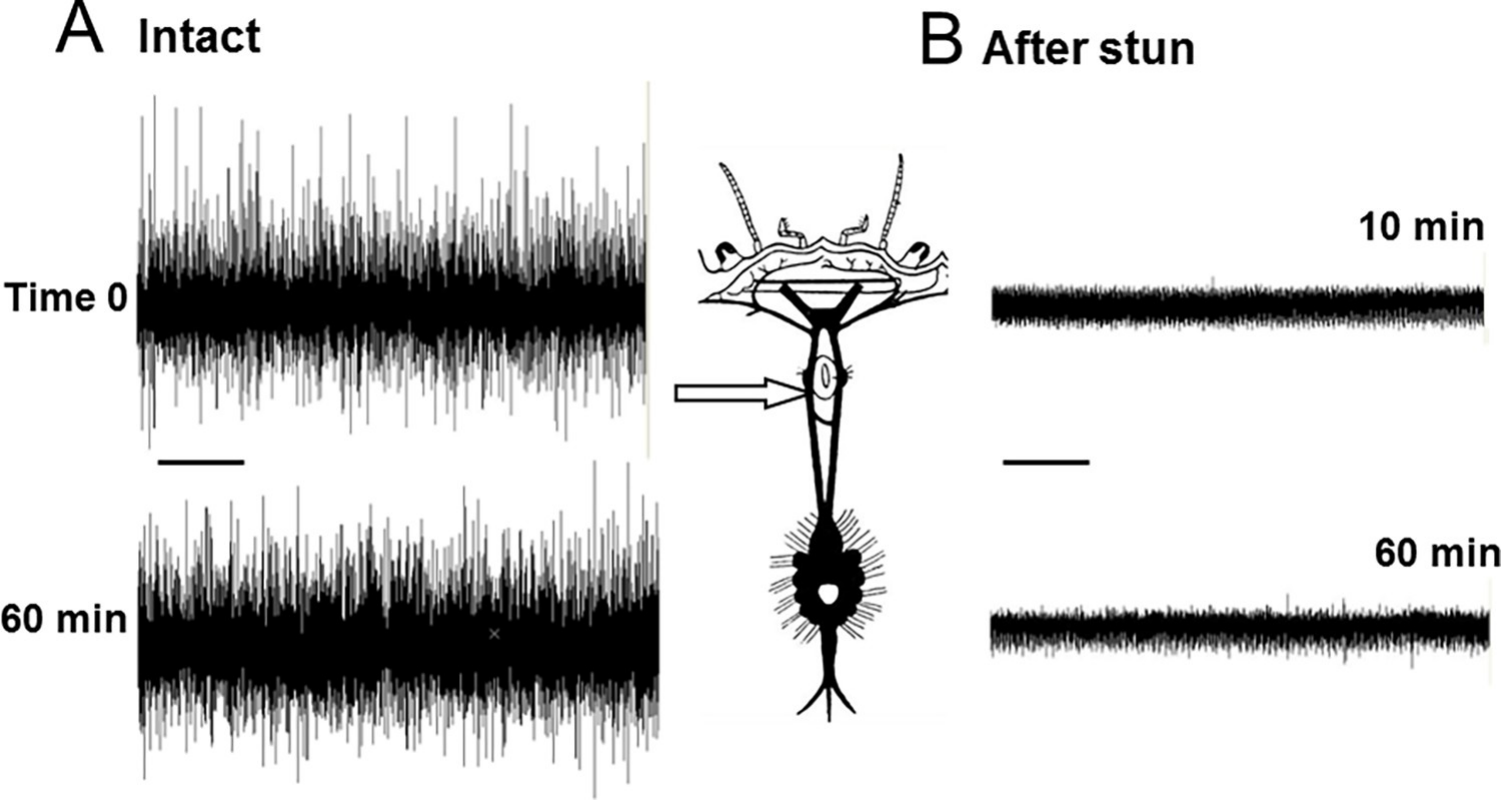
Time course of CNS activity with and without electrical stunning. Spontaneous nerve activity recorded extracellularly in the left circumoesophageal connective of *C*. *pagurus*. A: activity in intact animal initially and after 60 min; B: activity in another crab at 10 min and 60 min after electrical stunning. Scale bar 1s.

When tactile stimuli were applied to the eyestalks or antennae, there were systematic changes in firing frequency in some of these axons, indicating that these were conveying descending activity from the brain (Fig 3B). There were also high frequency bursts of activity that corresponded to the animal making struggling movements (fictive locomotion) (Fig 3C). By cutting the circumoesophageal connectives and making recording from above or below this point it was possible to confirm that there was both ascending and descending activity present (Fig 4A and 4B). In order to test the persistence of activity in the CNS, some preparations were re-tested at intervals. Data are shown for 60 min (Fig 5A), but activity actually continued for up to the longest time tested (4 hours).

Recordings from the central nervous system of crabs that had been subjected to electrical stunning indicated that no neuronal activity was detectable in the circumoesophageal connectives in any of the individuals at either 10 min (Fig 4C and 4D) or 60 min (Fig 5B) after stunning, or up to the longest time tested (4 h). Consistent results were obtained from all of the six crabs tested.

### Activity in the peripheral nervous system of intact crabs—sensory responses

**Fig 6:**
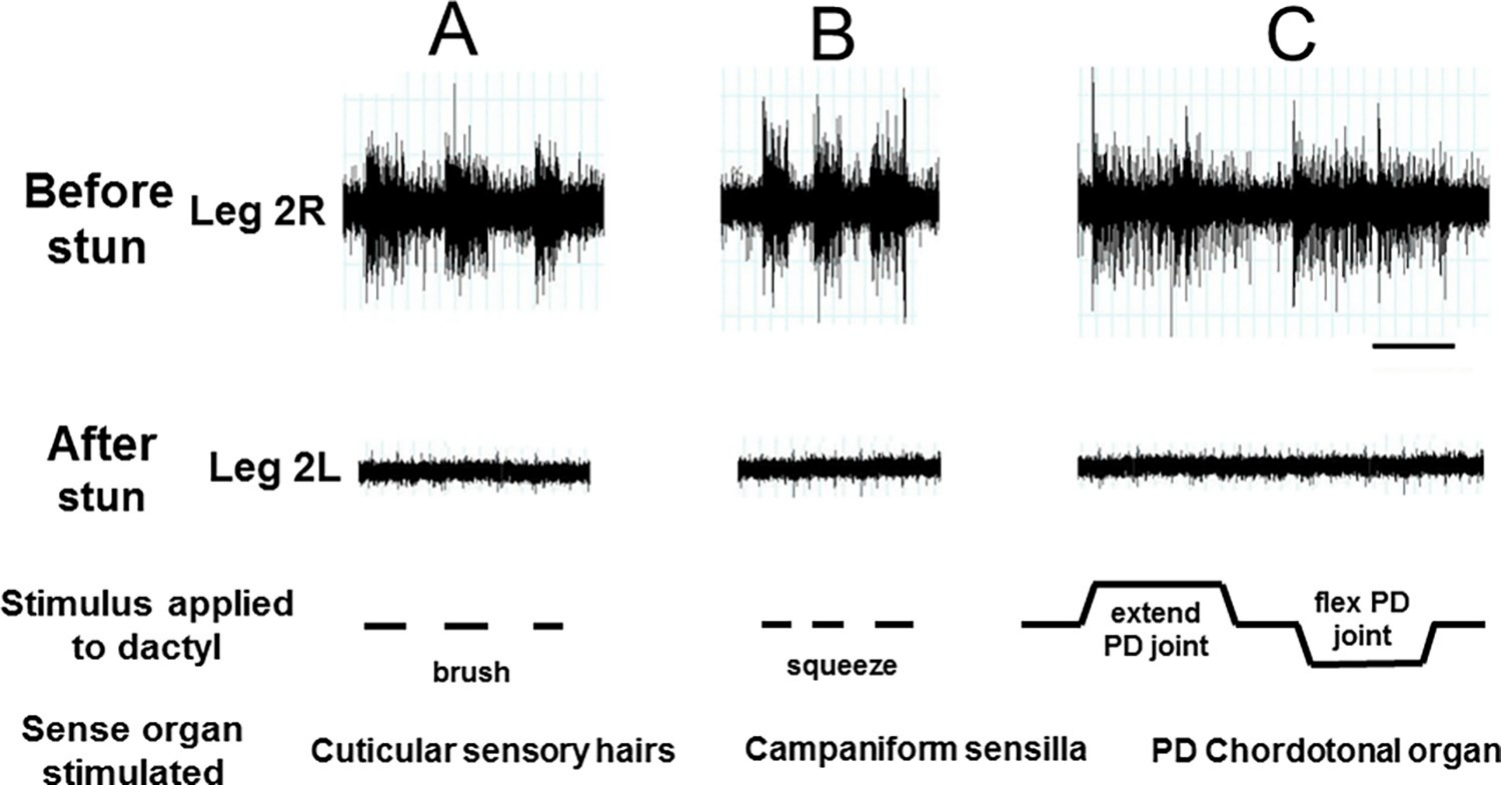
Recording sensory activity before and after electrical stunning. Responses of the leg nerve of *C*. *pagurus* preparation to three forms of stimulation of the dactylopodite (A-C) as indicated. Top panels, leg R2 autotomised from intact animal before stunning; lower panels, leg L2 amputated from same animal after electrical stunning. Scale bar 5 s.

**Fig 7:**
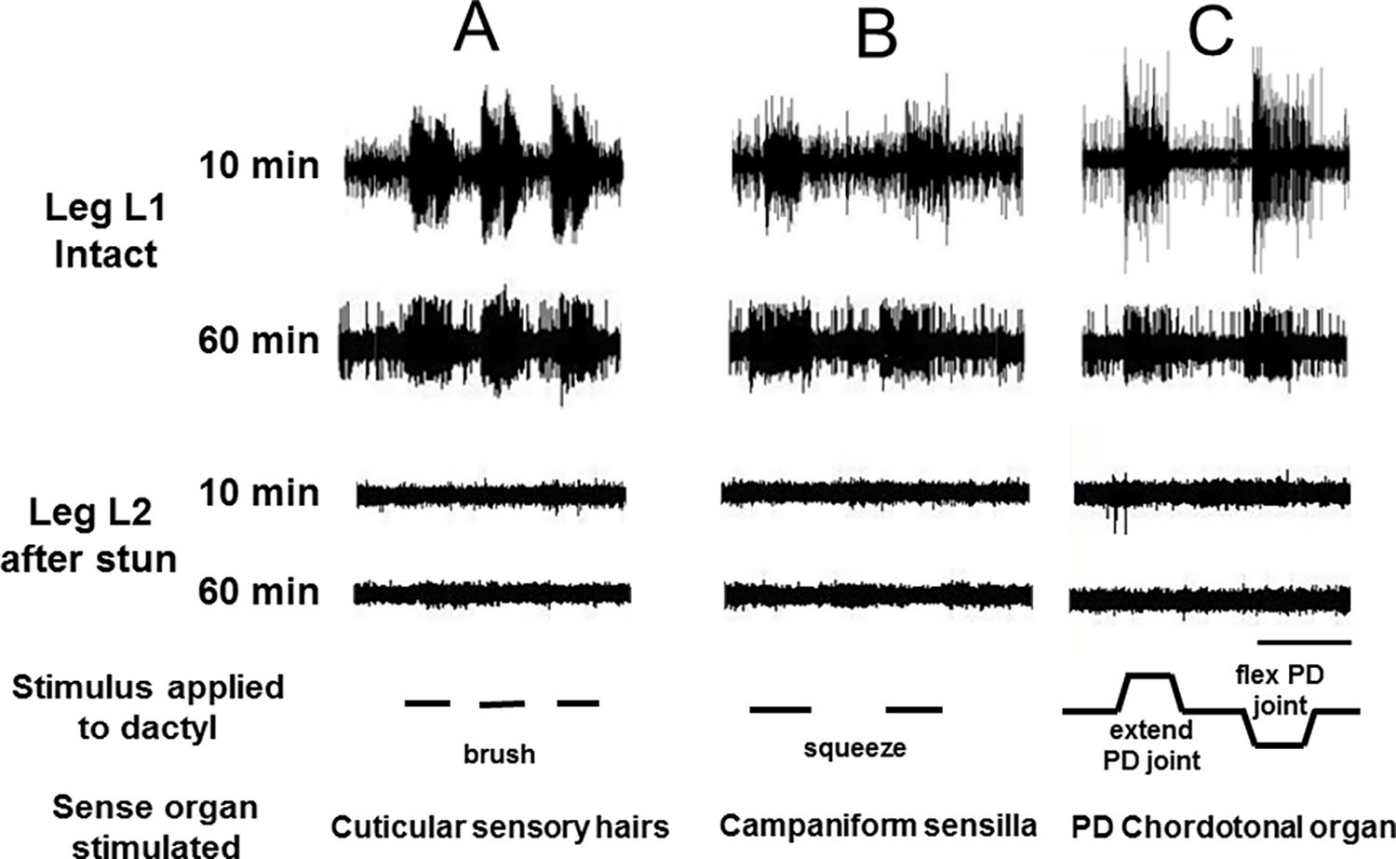
Time course of sensory activity with and without electrical stunning. Responses of sensory neurones in the leg nerve of the crab *C*. *pagurus* to three forms of stimulation of the dactylopodite (A-C) as indicated, measured at 10 min and 60 min after leg isolation. Top panels, leg L1 autotomised from intact animal; lower two panels, leg L2 amputated from the same animal after electrical stunning. Scale bar 5 s.

Activity in the peripheral nervous system in intact crabs was recorded from sensory neurones in isolated legs, following autotomy (Figs 6 and 7). The application of various stimuli to the distal part of the leg clearly elicited activity in a number of sensory neurones. Their patterns of activity were typical for the various sense organs that were stimulated in each case. Thus brushing movements over the cuticle of the dactylopodite produced bursts of activity typical of the responses to displacement of cuticular sensory hairs (Figs 6A and 7A, left panels). Compression (squeezing) of the cuticle of the dactylopodite elicited persistent tonic responses for the duration of the stimulus consistent with activation of campaniform sensilla (Figs 6B and 7B). Finally, the discharges to the movement and displacement phases of flexions and extensions applied at the P-D joint had the phasic and tonic components characteristic of chordotonal organ responses (Figs 6C and 7C).

In order to test the persistence of sensory responsiveness in the leg nerves of intact crabs, some isolated leg preparations were tested or re-tested at increasing time intervals after autotomy. Data are shown for 10 min and 60 min (Fig 7, upper two panels). The sensory responses obtained after these times (and up to 4 h) were as strong and of the same pattern as those recorded immediately after autotomy.

After electric stunning of the crabs, sensory activity in the nerves of virtually all the amputated legs tested was absent in response to all of the three stimuli applied (Figs 6 and 7, lower panels), and there was no apparent recovery of responsiveness at 60 min (Fig 7, lower pairs of panels) or up to the longest time tested (4 h).

### Motor activation of muscle contraction

**Fig 8:**
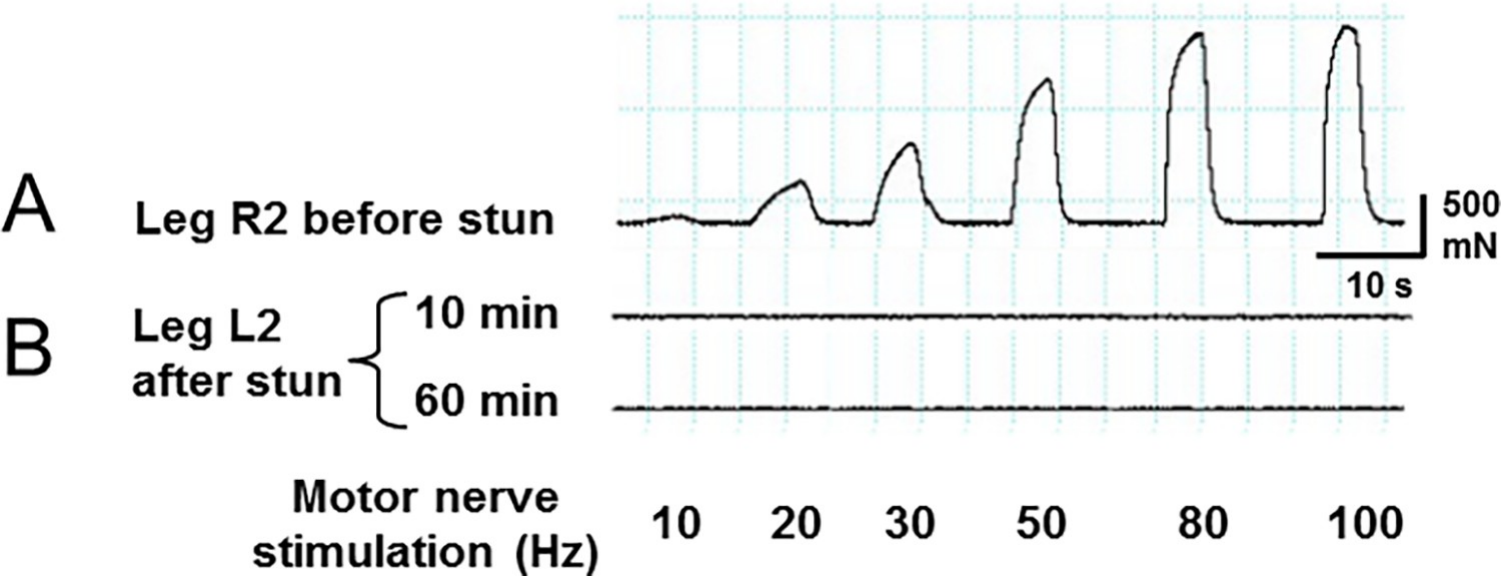
Recording motor output before and after electrical stunning. Forces produced by the dactylopodite closer muscle of the leg of *C*. *pagurus* in response to stimulation of motor neurones in the leg nerve at various frequencies. A: leg R2 autotomised from intact crab; B: leg L2 amputated from the same crab after electrical stunning, measured at 10 min and 60 min post-stun. Stimulus voltage 4V.

The normal operation of the motor pathways of the peripheral nervous system was demonstrated by stimulating the nerve of an autotomised leg from an intact crab at various frequencies while monitoring the force produced by the dactylopodite closer muscle. The force varied in a frequency-dependent manner typical of crustacean neuromuscular systems due to their synaptic properties of summation and facilitation (Fig 8A). After electrical stunning there was no detectable muscle force development in response to stimulating the motor nerves either 10 min or 60 min post-stun (Fig 8B), or up to 4 h.

### Stress measurement

**Fig 9:**
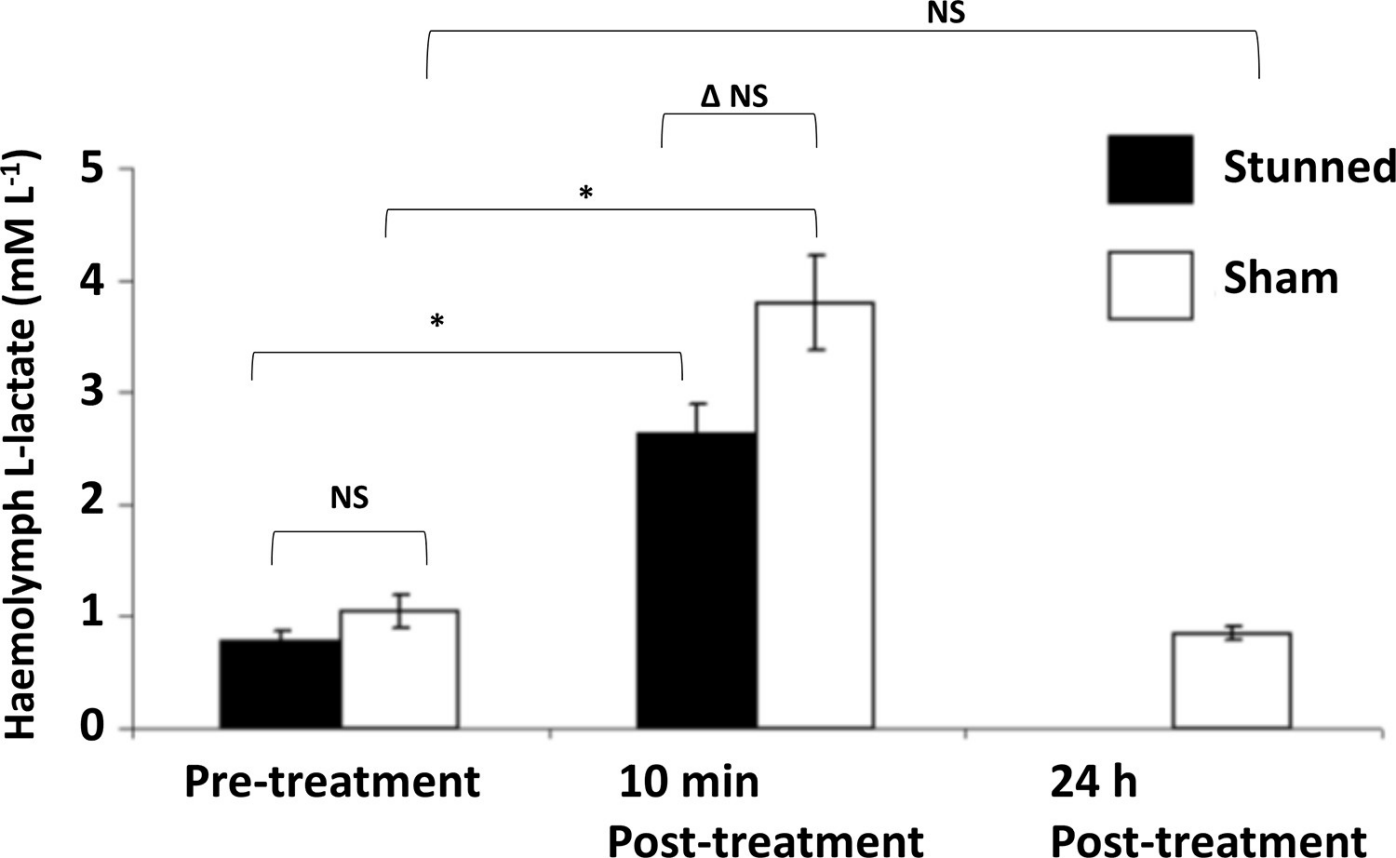
Measures of physiological stress before and after electrical stunning. Haemolymph L-lactate concentrations in *C*. *pagurus* before and after electrical stunning (black bars) or a sham-treatment (white bars). Mean values ± SEM. N = 6 for each treatment group. (*) indicates that values are significantly different (p<0.05), (NS) indicates values are not significantly different, (Δ) indicates the difference/increase following the two treatments considering the changes in the values for individual crabs; (t-test p<0.05).

The physiological stress associated with the electrical stunning process was assessed by monitoring L-lactate in the haemolymph (Fig 9). The haemolmph L-lactate in the two groups of 6 intact crabs taken randomly from the holding tanks for either electrical stunning or the sham treatment had mean values of 0.78 ± 0.09 mM L^-1^ (pre-stun) and 1.05 ± 0.15 mM L^-1^ (pre-sham) respectively. These values did not differ significantly from each other (F_1,11_ = 2.47, P = 0.147). Following electrical stunning the haemolymph L-lactate in the crabs increased to a mean value of 2.63 ± 0.26 mM L^-1^, which was significantly greater than the pre-stun value for this group (F_1,11_ = 45.00, P = 0.000). After the sham treatment the haemolymph L-lactate in the crabs also increased, with a mean value of 3.80 ± 0.42 mM L^-1^ being obtained. This was also significantly greater than the pre-sham value for this group (F_1,11_ = 38.49, P = 0.000).

The increases of haemolymph L-lactate following the two treatments were compared by considering the changes in the values for individual crabs, and it was found that the mean increase for the electrostunned crabs (1.85 ± 0.30 mM L^-1^) was not significantly different from the mean increase for the sham-treated crabs (2.75 ± 0.36 mM L^-1^) (F_1,11_ = 3.68, P = 0.084).

In terms of their subsequent fates, when returned to their holding tanks the electrically stunned crabs showed no further visible movements, and never recovered. However, the sham-treated crabs showed normal behaviour when returned to their holding tanks (limb movement, antennule flicking, a ventilation current and eye retraction reflexes) which continued thereafter. Samples taken from these sham-treated crabs 24 h later showed that they had a mean haemolymph L-lactate concentration of 0.85 ± 0.06 mM L^-1^, which was not significantly different from the pre-sham value of 1.05 ± 0.15 mM L^-1^ for this group (F_1,11_ = 1.64, P = 0.229).

## Discussion

The results presented here based on neurophysiological recordings provide a more reliable indication of the loss of sensibility in *C*. *pagurus* than can be ascertained from the observation of their behavior and movements alone. They therefore give greater insights into the effectiveness of electrical stunning, and suggest possible underlying causes.

### Activity in the nervous system

The results from crabs are consistent with the literature on the neurophysiology of crustacean nervous systems (see, for example, [59]) in showing that the central nervous systems of decapod crustaceans display continuous nerve activity, which in turn produces outputs in the motor nerves to the body and limb muscles. A characteristic feature that is common in decapod nervous systems is the long-lived nature of continued activity and signal conduction, even when isolated from the rest of the body [60–62]. It is widely reported that, provided the structures are bathed in an appropriate saline solution, activity can continue for many hours, and indeed this was observed in the present study both with the central nervous preparations and with the isolated crab legs after autotomy. Such robustness provides more certainty in interpreting any loss of activity following a procedure such as electrical stunning as being due to the intervention itself, rather than to any underlying general decline with time in nervous system activity or responsiveness.

### The use of autotomised legs

Autotomy is a natural process for defence [63], invoked by particular stimuli [57]. In decapod crustaceans it involves a specific neuromuscular reflex acting across a fixed cuticular plane of fracture [56], although there may be a degree of voluntary control [64]. In addition to specific mechanosensory stimuli, crabs show autotomy when an appendage has been subjected to various other stimuli such as heating, cooling on ice, shock, wounding, acetic acid injection or minor electric shocks to the appendages [65].

Induced autotomy was used in the present study to obtain an isolated leg from an intact crab, since it has been previously found that this process has virtually no measurable effect on the stress levels in the animal, as indicated by the low levels of L-lactate circulating in the haemolymph [42]. Forced removal of a limb in the living animals was not employed, since it is known to induce a significant stress, due to the extent of tissue damage imposed [42]. Limb amputation was only used on animals which had been subjected to the electrical stunning process and were judged to be dead.

### The effects of electrical stunning

Published studies of electrical stunning [22, 30, 31, 33] have used a variety of devices that produced various waveforms, voltages and currents. They found that after stunning large decapod crustaceans were effectively anaesthetised for a range of times (5 minutes to 40 minutes) but thereafter showed some signs of recovery (i.e a reversible stunning). However this is sufficient time for another method of dispatch to be applied while the animals are under this stunning anaesthesia. Our study found that crabs can both be irreversibly stunned and also killed by electrical stunning, thus qualifying as ‘simple stunning’ [6]. This finding is consistent with independent performance tests on the Crustastun™ with the same species of crab [16] that found that following a 10 s stun none of the crabs tested showed any sign of recovery over the period assessed (> 1h). This suggests that more research is needed to define the electrical parameters of stunners that optimize their ability to act as ‘simple stunning’ devices. Other considerations include the potential risk of heating the crab due to the current flow through its body, although this has been shown to be only localized to the carapace if the stun duration is no more than 10 s [33]. Moreover, for the commercial processing of large numbers of captured decapod crustaceans, an electrical stunner must have the capacity for a high throughput of animals while delivering effective stunning despite animals being of different sizes and in different positions relative to the electrodes. This will entail a re-design of single in-air/dry stunners such as the Crustastun™ to ones based on continuous flow or conveyer belt systems. Such systems exist for fish [25, 26], but are still at the prototype stage for crustaceans [33].

In terms of the effect of electrical stunning on nerve activity in crabs, our results are relatively conclusive within the constraints of the small sample size of animals examined. According to the extracellular recording method used, the various forms of spontaneous activity within the central nervous system were completely arrested. The recordings made on isolated crab legs allow some further conclusions to be drawn, namely that electrical stunning also has a specific effect on the functioning of the peripheral parts of the nervous system. There was both a loss of responsiveness to three types of sensory stimulation, and also a failure in neuromuscular activation. The first of these effects would have rendered the animals insensitive to external stimuli, while the second would have rendered them immobile.

Taken together these results provide the first indication that electrical stunning incapacitates the nervous system of *C*. *pagurus* simultaneously at two levels, centrally and peripherally, which completely prevents all normal neuronal functioning. The rate at which this happens cannot be ascertained directly from our experimental protocol, but two other studies provide some insights. Sparrey (in [16]) found that the threshold stunning current (1.3 amp) in the Crustastun™ that is able to stun these crabs into insensibility is achieved within 0.5 s, so the stunning effect must occur almost instantaneously. Fregin and Bickmeyer [27] studied electrostunning in lobsters by using implanted electrodes adjacent to the ventral nerve cord, and so were able to obtain records immediately after the termination of a 10 s stun with the Crustastun™. They found that a high-frequency intense neuronal discharge (seizure) occurs over the first minute post-stun that masks any responses to external stimuli, but that thereafter neuronal activity is seen to be unresponsive to sensory activation and in fact fades away (in their case for up to 1 h). A conservative estimate is therefore that effective stunning is accomplished within one minute of the termination of the stun cycle, while it is probable that it occurs even sooner.

The extracellular recording method used does not allow us to identify the mechanism whereby the afferent sensory signals and the efferent motor activity in the peripheral nervous system are suppressed. It is possible that the conduction processes in the axons of both the sensory and motor neurones have been disrupted by the electrical currents generated by the stunning cycle. However, it cannot be excluded that for the afferent side the electrical stunning has affected only the sensory transduction processes in the receptor endings of the sense organs, rather than the nerve transmission mechanism in the sensory nerves. Similarly, for the efferent side electrical stunning may have interrupted synaptic transmission at the neuromuscular junctions, and/or excitation-contraction coupling processes within the muscle fibres, rather than the nerve transmission mechanism in the motor nerves. It is of course possible that all of these processes have been affected simultaneously. To distinguish between these possibilities would require an examination of each of the contributing processes by using other, more appropriate, electrophysiological methods in a targeted approach.

Electrical stunning with the Crustastun™ did not induce autotomy of the claws or legs of the crab. This finding is consistent with results we have obtained using this instrument on other decapod species [29] and with the experience of operators using it routinely in commercial settings. It suggests that the observed inactivation of the sensory and motor divisions of the nervous system must have included the neuromuscular reflex pathways underlying autotomy. This is especially striking since attempts to electrically stun crabs using a low electrical field strength to the whole animal [30] failed to inactivate the animals, but actually caused extensive autotomy. Moreover, Elwood *et al*. (2009) found that weak electrical stimuli applied to the legs induced them to autotomise [65]. A plausible interpretation of these different findings is that weak electrical stimuli artificially activate the sensory and/or motor pathways involved in the autotomy reflex, resulting in the shedding of the limb, whereas electrical stunning with higher electrical field strengths inactivates these pathways rapidly and completely, before the reflex neuromuscular action underlying autotomy can be elicited, so that no limb losses occur.

### Physiological stress

The main findings were that there was a measurable effect of both electrical stunning and the sham treatment on the haemolymph L-lactate concentrations of *C*. *pagurus*, but that there was no statistically significant difference between the effects of these two treatments. This implies that the stress or muscular activity imposed during the stunning procedure was in fact no greater than that induced by the brief emersion (aerial exposure), the handling and the haemolymph sampling performed, that were common to both treatments. We therefore conclude that it is unlikely that electrical stunning process imparted additional stress.

It is relevant to consider what these levels of haemolymph L-lactate represent in absolute terms. This can be judged by considering the values of haemolymph L-lactate obtained here in relation to those obtained in other studies on this and other decapod crustacean species in response to a range of other stresses (Table 1). For *C*. *pagurus*, haemolymph L-lactate concentrations can reach much higher values when the animals are exposed to stresses such as emersion, handling and those associated with transportation. Values of 10.0 mM L^-1^ have been reported for *C*. *pagurus* after transportation [38], and indeed can reach more than double that value when this is combined with emersion at elevated temperatures [66]. Similarly, for the lobster *Homarus gammarus* a mean value of 12.5 mM L^-1^ was obtained following transportation [67]. The relative increases in the measures are also relevant. The increases in haemolymph L-lactate concentrations in *C*. *pagurus* from before to after electrical stunning or sham treatment represent around a 3.4-fold increase. These increases are an order of magnitude smaller than those induced by the most extreme stresses.

**Table 1:** Haemolymph L-lactate concentrations measured in decapod crustaceans in response to various stressors.

Species	Stress	Haemolymph L-lactate initially (mM L^-1^)	Haemolymph L-lactate after stress (mM L^-1^)	Reference
*Cancer pagurus*	Baseline values	1.89 ± 0.38	-	[69]
*C*. *pagurus*	Electrostunned	0.78 ± 0.09	2.63 ± 0.26	Present study
*C*. *pagurus*	Sham stun	1.05 ± 0.15	3.80 ± 0.42	Present study
*C*. *pagurus*	Transport in air (36 h)	0.34 ± 0.08	10.81 ± 1.12	[38]
*C*. *pagurus*	Transport in air (58 h)	1.92 ± 0.75	5.77 ±1.23	[66]
*C*. *pagurus*	Dry storage (18 h, 20° C)	2.2 ± 3.4	44.0 ±2.9	[70]
*Homarus gammarus*	Electrostunned	0.8	2.3	[71]
*H*. *gammarus*	Sham stun	0.7	1.9	[71]
*H*. *gammarus*	Transport	0.4	12.5	[67]
*Jasus lalandii*	Emersion	1.0	18.5	[72]
*Nephrops norvegicus*	Emersion	0.6	19.6	[72]
*N*. *norvegicus*	Trawling	-	12.0	[17]
*Liocarcinus depurator*	Trawling + emersion	-	14.7	[73]
*Orconectes limosus*	Emersion	-	19.7	[74]
*Gecarcoidea natalis*	Exercise	0.46	>20.0	[40]
*Chionoecetes opilio*	Emersion at 10° C	0.1	8.9	[75]
*Lithodes santolla*	Emersion at 8° C	<0.1	38.0	[76]

The return of haemolymph L-lactate concentrations in sham-treated crabs to pre-treatment resting levels within 24 h is as expected, and although the detailed time course of this was not documented, other studies suggest that it would to have taken several hours to subside following the imposed stress (see for example [17]). In contrast, since electrical stunning invariably led to the death of the animals, it was not relevant in those cases to continue measuring haemolymph L-lactate concentrations at later time points since the animals were then in a post-mortem state, and it is known that during this period there is an extensive anaerobic fermentation in the tissues, leading to a rapid production of large amounts of L-lactate [68]. This highlights the fact that the interpretation of L-lactate data as an indication of stress has to be made with caution, since they can reflect *in vivo* stress, muscle contraction, exhaustive exercise, or *post-mortem* processes, depending on the situation.

### Conclusions

It is concluded that electrical stunning with the Crustastun™ can rapidly arrest spontaneous activity within the central nervous system, with an accompanying loss of sensory responsiveness and a failure in neuromuscular activation. In effect this stunning induces rapid anaesthesia, which renders the animals insensible within 10 s. Moreover, as judged by indicative biochemical measures, it imposes no more physiological stress than does brief handling of the animal. This stun is also irreversible, leading to the death of all 18 of the crabs that were stunned. For all these reasons this procedure may meet the criteria for being a humane method of slaughter for *C*. *pagurus* [12, 16]. It remains to be confirmed whether this also applies to other crabs or other decapod crustaceans, both large and small, that are widely consumed seafood species. Certainly prior to application to other species the electrical parameters used will have to be tailored to suit the size, developmental stage and moult condition of the particular species to be stunned.
